# S115. SYMPTOM DOMAINS IN SCHIZOPHRENIA AND GENE POLYMORPHISMS

**DOI:** 10.1093/schbul/sby018.902

**Published:** 2018-04-01

**Authors:** Vjekoslav Peitl, Branka Vidrih, Draženka Ostojić, Tanja Franćeski, Milena Peitl, Dalibor Karlović

**Affiliations:** 1 University Hospital Center Sestre Milosrdnice, Catholic University of Croatia; 2 Psychiatric Clinic Vrapče; 3 University Hospital Centre Sestre milosrdnice; 4 University Hospital Centre Zagreb

## Abstract

**Background:**

Dopamine and serotonin neurotransmission relies mostly on the action of four factors: serotonin and dopamine transporters (SERT and DAT) and enzymes monoaminooxidase A (MAO-A) and catechol-O-mehtyltransferase (COMT). The goal of this research was to closely examine schizophrenia symptom domains in relation to the investigated polymorphisms.

**Methods:**

Study group was composed of 300 schizophrenic patients. Severity of schizophrenia was assessed by the Positive and negative syndrome scale (PANSS), depressive symptoms were assessed with Calgary depression scale for schizophrenia (CDSS). SERT (5-HTTLPR), DAT (VNTR), COMT (Val158Met) and MAO-A (VNTR) gene polymorphisms were analyzed. Schizophrenia symptom dimensions were determined with multivariate statistical methods, while logistic regression and ANOVA were used to investigate the influence of a genotype on a symptom domain.

**Results:**

Factor analysis of PANSS scale retained all 30 items and identified 5 separate factors (aggressive/impulsive, affective/depressive, cognitive, negative and positive symptoms). Analysis of CDSS scale revealed 2 separate factors (depression and suicidality).

Statistically significant PANSS variables were those of aggressive/impulsive and negative symptoms, while suicidality was the only significant CDSS variable.

**Discussion:**

Our PANSS scale factor analysis established 5 distinct factors. Previous factor analyses provided from 3, up to 7 different factors, but mostly 5 distinct ones: negative symptoms, positive symptoms, depressive symptoms, excitement and disorganization. That factor distribution corresponds to our findings in terms of identified number of factors, but seems to differ in terms of item distribution within those factors.

When testing the influence of investigated gene polymorphisms on the variable of total PANSS score and five distinct factors we did not establish significant findings regarding four variables: total PANSS score, positive, cognitive and affective/depressive symptoms. While that is in line with majority of other investigations, SERT promoter polymorphism and COMT Val158Met gene polymorphism have been previously associated with depressive and positive symptoms. SERT and MAO-A polymorphisms separately had a significant effect on the variable of aggressive/impulsive symptoms, which has not been reported earlier. Furthermore, significant influence of COMT gene polymorphism was established for the variable of negative symptoms, which is a confirmation of some earlier reports, although there have been contrary findings.

Previous reports of CDSS scale factor structure are limited to data from its initial validation to the few recent findings of its three-factor structure (depression, cognition and melancholy). We identified 2 separate factors using factor analysis, “depression” (which included seven out of nine items) and “suicidality”. To the best of our knowledge this is the first investigation of the putative association between any of the four investigated polymorphisms and depressive symptoms of schizophrenia measured by the CDSS scale. Ultimately, we did not establish a significant association of investigated gene polymorphisms and total CDSS score, as well as the “depression” factor. However, there was a significant association between the “suicidality” factor and SERT and MAO-A gene polymorphisms, as well as their interaction. The fact that the significant association was established for only one of the two obtained CDSS factors suggests that the association is subtle and, at least partially, explains rarely reported associations between investigated gene polymorphisms and schizophrenia symptom domains, which is especially true for depressive symptomatology.

